# Artificial Intelligence in Fluorescence Lifetime Imaging Ophthalmoscopy (FLIO) Data Analysis—Toward Retinal Metabolic Diagnostics

**DOI:** 10.3390/diagnostics14040431

**Published:** 2024-02-16

**Authors:** Natalie Thiemann, Svenja Rebecca Sonntag, Marie Kreikenbohm, Giulia Böhmerle, Jessica Stagge, Salvatore Grisanti, Thomas Martinetz, Yoko Miura

**Affiliations:** 1 Institute for Neuro- and Bioinformatics, University of Lübeck, 23538 Lübeck, Germany; 2 Department of Ophthalmology, University of Luebeck, University Hospital Schleswig-Holstein, Campus Lübeck, 23538 Lübeck, Germany; 3 Institute of Biomedical Optics, University of Lübeck, 23538 Lübeck, Germany

**Keywords:** fluorescence lifetime imaging ophthalmoscopy, artificial intelligence, support vector machine, smoking, retinal metabolism, small data

## Abstract

The purpose of this study was to investigate the possibility of implementing an artificial intelligence (AI) approach for the analysis of fluorescence lifetime imaging ophthalmoscopy (FLIO) data even with small data. FLIO data, including the fluorescence intensity and mean fluorescence lifetime (τm) of two spectral channels, as well as OCT-A data from 26 non-smokers and 28 smokers without systemic and ocular diseases were used. The analysis was performed with support vector machines (SVMs), a well-known AI method for small datasets, and compared with the results of convolutional neural networks (CNNs) and autoencoder networks. The SVM was the only tested AI method, which was able to distinguish τ_m_ between non-smokers and heavy smokers. The accuracy was about 80%. OCT-A data did not show significant differences. The feasibility and usefulness of the AI in analyzing FLIO and OCT-A data without any apparent retinal diseases were demonstrated. Although further studies with larger datasets are necessary to validate the results, the results greatly suggest that AI could be useful in analyzing FLIO-data even from healthy subjects without retinal disease and even with small datasets. AI-assisted FLIO is expected to greatly advance early retinal diagnosis.

## 1. Introduction

Fluorescence lifetime imaging ophthalmoscopy (FLIO) is a new diagnostic tool that measures the fluorescence lifetime (FLT) of retinal intrinsic fluorophores [1,2,3]. FLIO has been shown to indicate not only structural but also metabolic changes in the retina [4,5,6,7]. It utilizes a blue laser diode (473 nm) for the excitation of fluorophores, and the emitted photons are detected by two different detectors: the short spectral channel (SSC: for 498–560 nm) and long spectral channel (LSC: for 560–720 nm). The photons from the fluorophores, including flavin adenine nucleotide (FAD), macular pigment, collagen, elastin, and glycation end products (AGE), are considered to be predominantly detected in the SSC; and the fluorescence from A2E and lipofuscin, in the LSC [8].

Most recently, we reported a significant difference in the FLT between non-smokers and smokers in young healthy adults [9]. This result provides another strong implication that FLIO may be able to detect the metabolic state of the retina quite sensitively.

The FLIO data are shown as a pseudo color image of the FLT parameters at all measurement points (256 × 256 pixels) within a 30-degree field of view. The parameters include the mean FLT (τ_m_) as well as the values of its different components consisting of the FLT (τ_1,_ τ_2_…) and amplitude (α_1_, α_2_…). In the case of a morphologically healthy fundus, where disease-specific patterns are not apparently recognizable, it is very difficult to visually distinguish subclinical differences. A thorough statistical analysis might discover subclinical findings, but without a way to actively detect differences, it is difficult to apply them to clinical practice.

Furthermore, the FLIO evaluation requires precise knowledge of the complex data analysis, which is also time consuming. Establishing FLIO in everyday clinical practice would therefore benefit all the more from a simplified and accelerated data evaluation.

Therefore, we made an attempt to explore the feasibility of introducing artificial intelligence (AI) into the analysis of FLIO. In medicine, AI is playing an increasingly important role in supporting physicians in analyzing image data and making diagnoses [10]. In ophthalmology especially, the methods of machine learning already proved advantageous in the diagnosis of retinal diseases [11,12,13,14,15]. For example, Colomer et al. developed an algorithm for the detection of diabetic retinopathy in retinal fundus images [16], and Gallardo et al. were able to predict anti-VEGF treatment demands in patients with AMD, DME, or retinal vein occlusion [17]. While the studies mentioned above were able to access a sufficiently large datasets for the use of machine learning, one big problem in the clinical FLIO studies is the relatively low sample sizes for machine learning. There are many studies with less than 100 subjects [18,19,20,21], and also a few studies with less than 20 subjects per study group [7,22,23]. Therefore, a machine learning technique that might be able to also analyze small datasets would be desirable. Support vector machines (SVMs) belong to the group of supervised learning models that can learn from a given dataset to distinguish two “classes”, e.g., smokers and non-smokers, as in our study [24]. They are more robust for small datasets compared to other more complex machine learning algorithms [25]. There are already several studies that used SVMs successfully on datasets of about 50 to 75 subjects [26,27,28,29]. Yi et al., for example, were able to diagnose depression using an SVM on a dataset of only 55 subjects [27]. Thus, the aim of our study was to compare SVMs with other machine learning techniques as a new way of FLIO data analysis and to evaluate if there is a possibility of analyzing FLIO data with AI even with small datasets.

## 2. Materials and Methods

### 2.1. Clinical Dataset

The clinical dataset used for the current approach was obtained during our previous study [9], which we referred to for further insights into the clinical dataset. This previous study was a monocentric, prospective, cross-sectional clinical study at the Department of Ophthalmology of the University Medical Center Schleswig-Holstein, Campus Lübeck, where a total number of 54 participants, 26 non-smokers and 28 smokers, were enrolled between April 2021 and September 2021. For each participant, both eyes were measured, resulting in a total of 108 data points (samples). The study was positively reviewed by the ethics committee of the University of Lübeck and conducted in accordance with the ethical standards stated in the Declaration of Helsinki. The information about participants, general and ophthalmology data, can be referred to in that report. A summary of the demographic data is shown in Table 1, and a list of the data of all subjects is found in Supplementary Table S1.

Inclusion criteria for study participation were the ability to consent, healthy retinal findings, and subject age between 20 and 40 years. Exclusion criteria were subjects with retinal diseases, relevant media opacity, condition after eye surgery, and a narrow chamber angle that would not allow drug-induced mydriasis. No systemic diseases such as thyroid dysfunction or other hormonal disorders, diabetes mellitus, or hypertension were allowed to be present. Pregnant or lactating women were also excluded from the study. Based on previous literature [30], the group of smokers was defined as those who smoked five cigarettes daily for at least two years. In both groups, there were no significant differences in age or gender.

Given the assumption that differences between the two groups of non-smokers and smokers, if observable, could depend on the dosage of cumulative cigarette smoking, the smokers were further differentiated into two subgroups according to the number of cigarettes smoked during their lifetime. All smokers with a dose over the threshold of 2500 packs were labeled as heavy smokers, and those under this threshold as light smokers. This resulted in the 28 smokers being evenly grouped into 14 light and 14 heavy smokers. 

### 2.2. Data Acquisition for AI-Based Analysis

#### 2.2.1. Data from Fluorescence Lifetime Imaging Ophthalmoscopy (FLIO)

**Figure 1:**
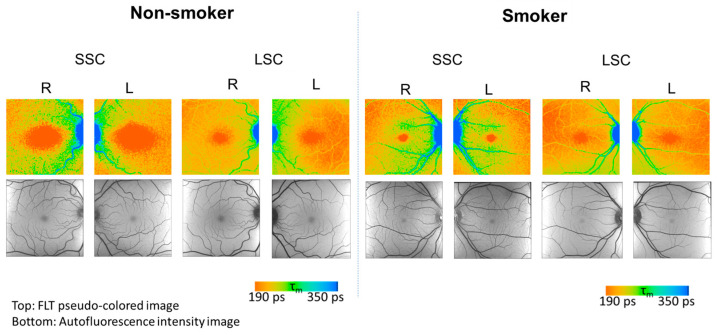
Representative FLIO data from a non-smoker (**left**) and a smoker (**right**). FLIO Data: Pseudo-colored images of mean fluorescence lifetime (τ_m_) (**top**) and intensity images (**bottom**). All show typical findings. The pseudo colors reproduce τ_m_ in a range of 190 to 350 picoseconds (ps; see color legend).

The detailed description of the FLIO method is described in our previous work [9]. Briefly, the FLIO original data consist of time-resolved photon counts at each pixel of fundus autofluorescence in the macula (30° × 30°, 256 × 256 pixels) (FLIO: Heidelberg Engineering, Heidelberg, Germany). For an analysis of the detected photon counts, the data were processed in the software SPCImage (version 8.0 NG, Becker&Hickl, Berlin, Germany) [31], where the obtained fluorescence decay was fitted to a biexponential function with a binning factor of 1. From this fitted function, the mean FLT (τ_m_) was calculated. These values are presented in pseudo color, as shown in Figure 1. For the AI analysis, the τ_m_ and the intensity data for all pixel positions from both spectral channels (SSC and LSC) from both eyes were exported as 256 × 256 matrix data. 

#### 2.2.2. Data from OCT Angiography (OCT-A) 

**Figure 2:**
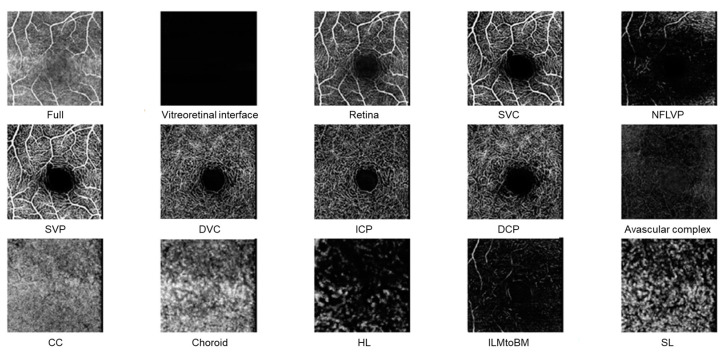
En face vascular images from the 15 default segmentations in OCT-A: Full: all layers, Vitreoretinal interface, Retina, SVC: superficial vascular complex, NFLVP: nerve fiber layer vascular plexus, SVP: superficial vascular plexus, DVC: deep vascular complex, ICP: intermediate capillary plexus, DCP: deep capillary plexus, Avascular complex, CC: choriocapillaris, choroid, HL: Haller’s layer, ILMtoBM40: internal limiting membrane to Bruch membrane, and SL: Sattler’s layer.

OCT-A (OCT2, Heidelberg Engineering) was performed for a macula region of 20° × 20° with a resolution of 512 × 512 pixels. The en face vascular images from the 15 default segmentations (Full, Vitreoretinal interface, Retina, SVC: superficial vascular complex, NFLVP: nerve fiber layer vascular plexus, SVP: superficial vascular plexus, DVC: deep vascular complex, ICP: intermediate capillary plexus, DCP: deep capillary plexus, Avascular complex, CC: choriocapillaris, Choroid, HL: Haller’s layer, ILMtoBM40: internal limiting membrane to Bruch membrane, SL: Sattler’s layer; Figure 2) were used for further analysis. One dataset had to be excluded from the analysis because of an error in the measurement angle. As a result, the final number of OCT-A datasets for the light smokers was 13 instead of 14.

### 2.3. Data Analysis Using Different AI Methods

#### 2.3.1. Preparation of FLIO Data

**Figure 3:**
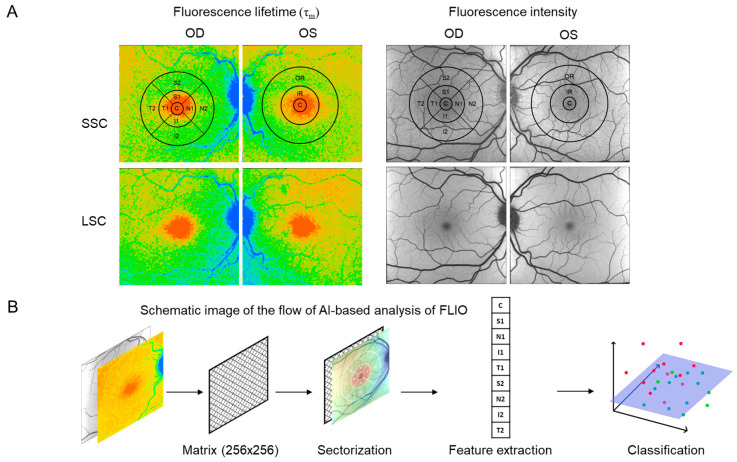
Processing of FLIO data for AI-based analysis. (**A**) For every grid section, the mean fluorescence lifetime (τ_m_) and fluorescence intensity value were computed for the extraction of features according to the grid of the early treatment diabetic retinopathy study (ETDRS), with the rings of the central area (C), inner ring (IR), and outer ring (OR), and the further division into the 4 subareas: nasal (N), superior (S), temporal (T), and inferior (I). (**B**) Schematic of the flow of the AI-based analysis of FLIO data. The workflow first obtains the data as 256 × 256 matrices for fluorescence intensity and fluorescence lifetime measurements for both the SSC and LSC, then obtains the means over the sectors of the ETDRS grid (sectorization), combines the means of all four matrices into one vector, and finally learns a classification with an SVM based on all data points from the dataset.

The FLIO data (matrix data of τ_m_ and fluorescence intensity value) were preprocessed using an integrated early treatment diabetic retinopathy study (ETDRS) grid, with rings of a central area (C), inner ring (IR), and outer ring (OR) with diameters of approximately 1 mm, 3 mm, and 6 mm. The ETDRS grid was centered over the fovea. Furthermore, the IR and OR were divided into 4 subsections: nasal (N), superior (S), temporal (T), and inferior (I) (Figure 3A). During a FLIO measurement, the subject must look at the cross-shaped central target, the center of the image (matrix data) was taken as the position of the central fovea (C).

#### 2.3.2. Initial AI Experiments with FLIO Data

**Figure 4:**
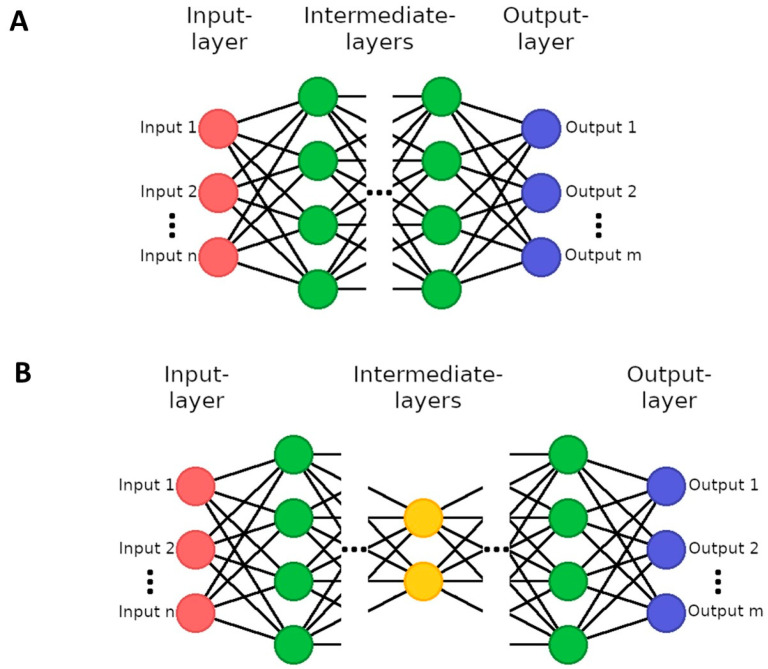
Schematics of (**A**) a general artificial neural network (ANN) and (**B**) an autoencoder network. The input layer matches the size of the input data to the neural network. ANNs can then have several intermediate layers with autoencoders typically having a central layer that is small compared to the other layers (a bottleneck). For classification tasks, the output layer matches the number of classes. For autoencoders, the output layer matches the size of the input layer.

For every grid section, the τ_m_ and fluorescence intensity value were computed for the extraction of features (Figure 3B). As a result, each FLIO measurement was represented by 9 values for both the τ_m_ and fluorescence intensity in each spectral channel, resulting in a total of 36 values. Initially, it was attempted to use convolutional neural networks (CNNs) to classify the FLIO data into the two groups of non-smokers and smokers. CNNs are a particular type of artificial neural networks (ANNs) (Figure 4A) that are inspired by how the human brain processes information. ANNs can take a numerical input tensor and process it into another numerical output tensor. They are typically trained on labeled datasets, where the network is rewarded for predicting the correct label and punished for predicting an incorrect label. Through this process, ANNs can learn to categorize data by extrapolating these to new data that it has not seen before and, for example, can then predict the probability that an image contains a certain kind of content. The special feature of CNNs is that they mimic simple and complex cells of the visual cortex, which is why they are very well suited for the analysis of images [10]. For these experiments, a ResNet18 model was used [32] and trained on 80% of the FLIO samples available, and then evaluated on the remaining 20%. 

Furthermore, it was attempted to acquire an information rich representation vector by using autoencoder networks (Figure 4B). Autoencoders have the purpose of predicting their own input as the output. However, this process involves downsampling the input to a very dense representation from which the original input is then recovered. Through this process, the autoencoder is encouraged to learn a very dense information representation of its input during the training process. In this work, we used a simple convolutional autoencoder with an encoding size of 50,176 and a U-Net autoencoder with an encoding size of 1024 [33].

#### 2.3.3. Preparation of OCT-A Data and t-Distributed Stochastic Neighbor Embedding

The OCT-A data were processed in two different ways: firstly, for each sample (each segmentation data as shown in Figure 2), a histogram of the pixel values, which range from 0 to 255, was calculated. This resulted in a 256-dimensional feature vector with each dimension corresponding to the abundance of a pixel value in the image. Secondly, each sample was encoded using an ImageNet [34] pretrained ResNet-50 [32] resulting in a 2048-dimensional feature vector. For both methods, these feature vectors were then reduced to their 15 most variable dimensions through a principal component analysis (PCA) [35] in order to reduce the size of the feature vectors and therefore the computational complexity of the final processing step. The result of the PCA was then further reduced to two dimensions as a t-distributed stochastic neighbor embedding (t-SNE) [36]. A t-SNE is a statistical method for embedding high dimensional data in a low (two- or three-) dimensional space while preserving a low distance between similar data points and a high distance between dissimilar data points. The t-SNEs were then inspected manually.

**Figure 5:**
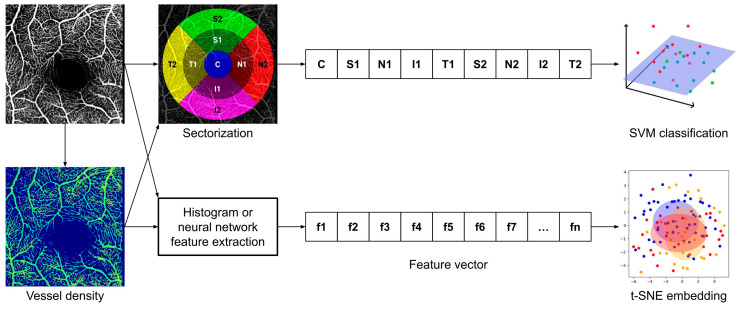
Schematic of the flow of the AI-based analysis of OCT-A. The workflow starts with the OCT-A image data. For some experiments, vessel density maps were calculated using local fractal dimensions. The data were then further processed using the means over the sectors of the ETDRS grid (sectorization) and learning classifications using SVMs or calculating histograms over the measurements or using neural networks to obtain feature vectors that were then compared between smokers and non-smokers using t-SNEs. All four possible combinations were executed for each analyzed sample.

For the second method, the images of the OCT-A data were sectorized using a circular grid of nine sectors similar to the ETDRS grid but adjusted in size to fit the region of measurement (Figure 5). The features obtained from this process were then analyzed with the same support vector machine (SVM) classification framework developed for the FLIO data (described below).

#### 2.3.4. Local Fractal Dimension of OCT-A Data

The local fractal dimension is a pixelwise measure of the roughness of an image that can be used to estimate the vessel density in OCT-A images. Using the method described by Gadde et al., vessel density maps were generated from the superficial vascular complex slab of each sample [37]. Using these density maps instead of the regular OCT-A images, the analysis methods of OCT-A data mentioned above were repeated.

#### 2.3.5. Support Vector Machine (SVM) for FLIO and OCT-A Data

Different subsets of the values from FLIO as well as from OCT-A were used as data to train and evaluate an SVM. The subsets were chosen to gradually narrow down the features that are most suited to distinguishing smokers and non-smokers, e.g., by starting with all features, comparing intensity vs. lifetime features, SSC vs. LSC, etc. SVMs are classification models that can learn, as mentioned before in the introduction, from a given dataset to distinguish two “classes” [24] and are also quite robust for small datasets [25]. Furthermore, using the kernel trick, SVMs can also learn non-linear classification functions, allowing for the separation of more complex data [38]. In this work, an SVM with a non-linear radial basis function kernel from the scikit-learn Python library was used [39].

For the evaluation, the results were averaged over 20 iterations of a 5-fold cross validation. This means that the data were randomly split into five groups such that the ratio of non-smokers, light smokers, and heavy smokers is preserved as well as possible (9–12 subjects each). Then, the SVM was trained on a combined four of these groups and tested on the fifth. This means that the SVM was trained on 42 to 45 subjects and tested on the other 9 to 12. For each subject, the measurements for both eyes were included in the same subset (training or test), resulting in a total of 84 to 90 training and 18 to 24 test samples for the SVM. The reason for including both eyes in the data was to introduce further variance since the measurements for both eyes of a subject are similar but not identical. This allows the model to generalize better from the training data. The 20 × 5 training runs were conducted independently with a new model being trained each time, and the average of the testing results was taken. 

### 2.4. Two-Sample T-Test

To find the most significant features out of the ETDRS sectorization feature vectors used in the SVM, a two-sample T-test was used on the τ_m_ values between the non-smoker and smoker groups for each combination of an ETDRS sector and spectral channel regarding fluorescence intensity or lifetime data, i.e., for each combination of sector, channel, and type of measurement, smokers and non-smokers were compared. For this purpose, the features with the highest significance in difference between the two groups were chosen.

## 3. Results

### 3.1. FLIO

#### 3.1.1. AI-Assessment with CNN and Encoder Networks

The CNN was unable to converge to a usable classifier, and the autoencoder applied was unable to learn the distribution of the FLIO data or to reproduce the input image, at least for such a small dataset, so these experiments were discontinued.

#### 3.1.2. AI-Assessment with SVM

The results of the classification of non-smoker and smokers for different subsets of the 36 features of lifetime (τ_m_) and intensity obtained from the ETDRS grid with SVMs are shown in Table 2. Firstly, looking at the subsets of FLIO including only fluorescence intensity and only τ_m_, the classification with the 18 intensity features shows an accuracy of approximately 48% for the differentiation between the eyes of smokers and non-smokers, effectively random chance. The 18 lifetime features, on the other hand, show an accuracy of 61%.

Follow up experiments investigating the τ_m_ in spectral channels show that using features only from the SSC also yields no effective differentiation of non-smokers and smokers while features from the LSC show an accuracy of 61%. Experiments on the spatial distribution show that features from the inner ring allow for a better accuracy (67%) than features from the outer ring (56%). Combining spatial and spectral channel features—the SSC features of the inner ring and LSC features of the outer ring and vice versa—shows the former achieving similar results as the inner ring features of both channels (65%) and the latter being at random chance (48%). Finally, picking the three features with the highest pairwise difference as indicated by a T-test yields the temporal and superior sectors of the inner ring for the SSC and the superior sector of the outer ring for the LSC. With these, the highest accuracy of 71% is achieved.

Based on the assumption that the effects of smoking may accumulate, these experiments were repeated for the same subsets to classify non-smokers and smokers with a lifetime cumulative smoking of more than 2500 packs (heavy smokers). The number 2500 is the median lifetime smoking (packs) of smokers in the study (Table 1). As shown in Table 3, for the subsets of only fluorescence intensity and only τ_m_ features, an accuracy of only 58% was observed for the 18 intensity features, which is again close to random chance. The 18 features on τ_m_, on the other hand, show an accuracy of 72% with approximately half of the heavy smokers being recognized (true positive rate (TPR) = 51%) and only about one in six non-smokers being falsely identified as a smoker (false positive rate (FPR) = 16%). For this reason, only lifetime features were used in further experiments.

Using the nine features from each spectral channel respectively shows a reduction in accuracy with the SSC features, achieving an accuracy of only 54%; and the LSC features, 67%, which is better than the SSC but still lower than both channels combined. In picking the eight features from either the inner or outer ring of the ETDRS grid but using both spectral channels, accuracies similar to those of using the full set of lifetimes features are achieved, 71% and 66%, respectively. However, using both rings but only the data from one spectral channel each yields the highest observed accuracy of 80% when using SSC features from the inner ring and LSC features from the outer ring. Conversely, using LSC features from the inner ring and SSC features from the outer ring results in a low accuracy of only 62%. Finally, an accuracy of 80% could be matched by providing the SVM with only 3 features per sample by picking the 3 features with the most significant pairwise differences among the 18 lifetime features as indicated by a T-test.

### 3.2. OCT-A

**Figure 6:**
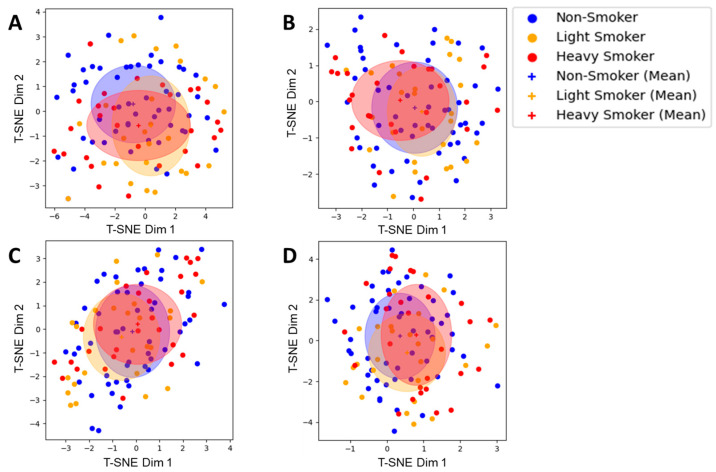
t-distributed stochastic neighbor embedding (t-SNE) plots of different image encodings with the means per group (+) and standard deviations (ovals) of OCT-A data. (**A**) Histogram encoding; (**B**) Neural Network encoding; (**C**) Sectorization encoding; (**D**) Sectorization encoding on a density map. The axes comprise the two dimensions (Dim 1, Dim 2) of the embedding space of the t-SNE and are therefore dimensionless and only describe two directions, where similar points from the origin space are clustered and dissimilar points are far from each other.

When evaluating the encodings of the whole images via histograms, neural networks, and sectorizations as t-SNE plots no significant difference between the groups of non-smokers and smokers—neither all smokers nor only heavy smokers—could be observed (Figure 6A–C). This was true for all three of these methods as well as for all investigated OCT-A layers. This was also true for the features of the density maps from a local fractal dimension analysis (Figure 6D).

Additionally, the features obtained through the sectorization of the images were evaluated with the same SVM classification framework that was previously used with the FLIO data. Here, no significant results could be observed except for features from the DCP layer where smokers could be classified with an accuracy of 68%, slightly surpassing the accuracy achieved when training the SVM on the full dataset (Supplementary Table S2). However, as opposed to the results with the FLIO data, reducing the dataset to non-smokers and heavy smokers did not yield an increased accuracy (Supplementary Table S3). Another follow-up experiment where only light smokers were attempted to be differentiated from non-smokers was unsuccessful too, with both TPRs and FPRs being close or equal to zero (Supplementary Table S4).

Finally, using the vessel density maps calculated using the local fractal dimension, no significant differences between non-smokers and smokers or heavy smokers could be observed at all. 

## 4. Discussion

AI is finding greater application in medicine, as it can support humans in areas that are highly demanding for them but that can be processed by artificial intelligence in record time. These include, in particular, the analysis of large and complex datasets and the discovery of relationships in these datasets. Most recent studies on the application of AI on medical image data have relied on training neural networks on thousands to hundreds of thousands of training samples [40,41,42,43].

Neural networks excel at finding patterns in data and generalizing from them; however, this often requires large datasets and even more so with larger networks. This is mostly because natural biological variances need to be learned, which might be minor differences in images but could have a large impact on the results. Furthermore, neural networks need to learn how to extract the relevant data from the images. FLIO is a very special domain compared to typical image classification because the data comprise pixelwise measurements of the fluorescence lifetime. Furthermore, since it is a relatively new imaging technique and most datasets are small, no other datasets of similar measurements are available for transfer learning; the common approach of using a model that has been previously trained on a different but structurally similar dataset could also not be used.

Therefore, in this work, we had only a very small dataset of 108 samples (both eyes) from 54 subjects available. This made the training of even standard neural networks such as CNNs unfeasible. SVM classifiers, on the other hand, are much less complex, and their inherent regularization schemes are designed for small datasets, so the amount of data required for training does not have to be so large [44], and SVMs could therefore successfully be applied to this task. Furthermore, this work used handcrafted features, reducing the 256 × 256 pixel images to vectors of 36 values. This circumvented the need to learn feature extraction using a large dataset. With these two strategies, promising results could be achieved even from this very small dataset. 

However, it could still be questioned whether such a small dataset is nevertheless sufficient, due to its size and since no validation with a second independent dataset has taken place. In order to validate the results, we would like to add that the results of the AI evaluation agreed with the statistical results obtained by us without the help of AI, where we also found significant differences between the two groups, most notably in the inner ring of the SSC and in the outer ring of the LSC [9]. Additionally, the risk of bias was reduced by having age-matched groups and by averaging over 20 iterations of a five-fold cross validation. Numerous pilot studies with FLIO on small datasets have already shown that FLIO can reveal significant differences even with small datasets [7,22], which is most likely related to the high sensitivity of FLIO.

We therefore believe that the small dataset is not a problem for our AI method or a problem with the implementation of AI in FLIO analysis, but that the small dataset should simply lead to some overall caution in the interpretation of the FLIO data, regardless of the use of AI in the analysis. 

Further examinations on larger datasets should be conducted for all FLIO results as it is a relatively new diagnostic instrument, but nevertheless, every result helps us a bit further to fully understand the method of FLIO and to simplify and accelerate its analysis.

Using the FLIO dataset of non-smokers and heavy smokers only for training and evaluation improved the accuracy of the SVM classifier compared to using the full set of smokers. This indicates that characteristics of the FLIO measures that depend on the amount of smoking were detected in the data. The results further suggest the fluorescence lifetime measurements to be much more relevant in this regard than the fluorescence intensity. These results are consistent with an earlier study about hydroxychloroquine toxicity, where FLIO was able to show changes in mild toxicity, whereas autofluorescence intensity images revealed no signs [45]. As this study’s and our previous study’s results both [9] indicate that smokers show a significantly longer τ_m_ in the inner ring of the SSC and a significantly shorter τ_m_ in the outer ring of the LSC, this combination is considered to be most suitable for examining the effects of smoking on the retina. Further expansion of FLIO analysis with AI including the single FLT components (τ_1,_ τ_2_…) will be part of future work. Furthermore, using data before data processing (fitting) is another option to be attempted. While this may complicate AI processing, it is practically attractive because it would eliminate one step of data processing in clinical practice.

The analysis of the OCT-A images did not show a strong differentiation between non-smokers and smokers except for data from the DCP layer, when analyzed with the SVM classification network on the feature vectors from image sectorization. However, follow-up experiments where the data were reduced to only light or strong smokers did not show such a differentiation capability for this layer. This suggests that some other property of this data, which is not related to smoking, might have induced a difference in the DCP layer between non-smokers and smokers in the initial experiment, which in turn could be attributed to the nature of the dataset being very small. The results from the vessel density maps of the OCT-A images showed that from this method no further information is obtained that can be utilized to separate non-smokers and smokers in the embedding space created by the encodings tested in this study.

Earlier studies on OCT-A in smokers revealed changes in vessel density [46] and in retinal blood flow using doppler velocimetry [47]. However, no differences in OCT-A were observed in our study. Perhaps this is due to the younger age of our study participants. As far as can be seen from this analysis, there were no obvious differences in blood flow between the two groups, further strengthening the possibility that the differences observed in FLIO between smokers and non-smokers in this study are related to metabolic changes or other alterations, rather than to changes in blood flow in the retina or choroid, as we already discussed in our previous study [9]. A different result may be obtained by examining the effects of smoking in older age groups, as the effect of long-term smoking is assumed to correlate more strongly with alterations in blood flow. 

A limitation of this study is, as mentioned above, the small sample size, which needs to be validated by studies with larger cohorts. In addition, it is not known whether the AI method used in this study is applicable to different variations of FLIO data. Additionally, this study was conducted on young, healthy subjects with no underlying disease. Clearly, much work remains to be conducted to ensure a comprehensive analysis that takes into account other factors such as age and underlying disease.

## 5. Conclusions

In conclusion, we have demonstrated the feasibility of implementing AI on FLIO data. In the case of novel diagnostic methods, the standard methods of training AI using a large amount of data are not feasible due to the small number of cases. In such a situation, an approach is needed that explores and develops methods to implement AI while increasing the number of cases. The SVM seems to be a good option to overcome this problem. Nevertheless, it is obvious that verification with larger datasets is still needed in the future.

Our goal is to explore how AI can be implemented in the new diagnostic method FLIO, so that it can be used in the future for the early detection of disease and determination of treatment efficacy. We believe that this work is a valuable first step toward this goal. 

## Figures and Tables

**Table 1:** Summary of the demographic data of all subjects.

Parameter	Unit	Non-Smokers (*n* = 26)			Smokers (*n* = 28)		
Male	(No. of subjects)		13			15	
Female	(No. of subjects)		13			15	
		Mean (SD)	Median	IQR	Mean (SD)	Median	IQR
Age	years old	26.7 (4.1)	26.5	23.0 to 30.3	28.5 (4.7)	28	25.0 to 32.0
Years smoked	years	0	0	0	12.0 (4.9)	10.8	9.0 to 15.8
Cumulative packs		0	0	0	2915 (2224)	2594	883 to 4280

(IQR: interquartile range).

**Table 2:** Results of SVM: Classification of non-smoker vs. smoker **(bold: Mean accuracy ≥ ~65%)**.

Feature Set	*n*	Mean TP	Mean FN	Mean FP	Mean TN	Mean TPR		Mean FPR		Mean Accuracy	
All features	36	32.70	23.30	21.35	30.65	58.39%	±6.44%	41.06%	±4.44%	58.66%	±4.63%
FLIO intensity	18	27.30	28.70	27.90	24.10	48.75%	±5.18%	53.65%	±6.20%	47.59%	±3.30%
FLIO τ_m_ only	18	34.90	21.10	21.15	30.85	62.32%	±4.85%	40.67%	±4.48%	60.88%	±3.14%
FLIO τ_m_; SSC	9	33.50	22.50	33.40	18.60	59.82%	±3.50%	64.23%	±5.35%	48.24%	±3.26%
FLIO τ_m_; LSC	9	33.25	22.75	18.90	33.10	59.38%	±6.00%	36.35%	±4.75%	61.44%	±3.91%
**FLIO τ_m_; IR**	**8**	**38.80**	**17.20**	**18.05**	**33.95**	**69.29%**	**±4.78%**	**34.71%**	**±5.91%**	**67.36%**	**±3.04%**
FLIO τ_m_; OR	8	27.25	28.75	19.05	32.95	48.66%	±3.33%	36.63%	±4.73%	55.74%	±2.68%
**FLIO τ_m_; IR- SSC, OR- LSC**	**8**	**35.00**	**21.00**	**17.05**	**34.95**	**62.50%**	**±3.66%**	**32.79%**	**±5.62%**	**64.77%**	**±3.54%**
FLIO τ_m_; OR- SSC, IR- LSC	8	26.80	29.20	27.45	24.55	47.86%	±5.43%	52.79%	±7.36%	47.55%	±3.24%
**FLIO τ_m_; T1-SSC, S2-LSC, S1-SSC**	**3**	**40.65**	**15.35**	**16.40**	**35.60**	**72.59%**	**±3.88%**	**31.54%**	**±4.49%**	**70.60%**	**±2.36%**

τ_m_: mean fluorescence lifetime, n: number of features, SSC: short spectral channel, LSC: long spectral channel, TP: true positive, FN: false negative, FP: false positive, TN: true negative, TPR: true positive rate, FPR: false positive rate, IR: inner ring, OR: outer ring, T1: temporal in the inner ring, S1: superior in the inner ring, S2: superior in the outer ring (ref. Figure 3A).

**Table 3:** Results of SVM: Classification of non-smoker vs. heavy smoker **(bold: Mean accuracy ≥ ~75%)**.

Feature Set	n	Mean TP	Mean FN	Mean FP	Mean TN	Mean TPR		Mean FPR		Mean Accuracy	
All features	36	11.85	16.15	9.50	42.50	42.32%	±7.34%	18.27%	±2.75%	67.94%	±3.02%
FLIO intensity	18	8.15	19.85	13.45	38.55	29.11%	±8.70%	25.87%	±3.67%	58.38%	±3.97%
FLIO τ_m_ only	18	14.30	13.70	8.45	43.55	51.07%	±3.41%	16.25%	±3.25%	72.31%	±2.72%
FLIO τ_m_; SSC	9	6.05	21.95	14.50	37.50	21.61%	±7.78%	27.88%	±3.96%	54.44%	±3.43%
FLIO τ_m_; LSC	9	11.75	16.25	10.15	41.85	41.96%	±5.16%	19.52%	±4.00%	67.00%	±2.60%
**FLIO τ_m_; IR**	**8**	15.30	12.70	10.70	41.30	54.64%	±6.40%	20.58%	±2.28%	70.75%	±2.72%
FLIO τ_m_; OR	8	11.95	16.05	10.95	41.05	42.68%	±6.03%	21.06%	±5.52%	66.25%	±3.49%
**FLIO τ_m_; IR- SSC, OR- LSC**	**8**	**18.05**	**9.95**	**6.40**	**45.60**	**64.46%**	**±5.23%**	**12.31%**	**±4.19%**	**79.56%**	**±3.31%**
FLIO τ_m_; OR- SSC, IR- LSC	8	9.15	18.85	11.90	40.10	32.68%	±5.90%	22.88%	±5.47%	61.56%	±3.75%
**FLIO τ_m_; T1-SSC, S2-LSC, S1-SSC**	**3**	**19.10**	**8.90**	**7.10**	**44.90**	**68.21%**	**±6.07%**	**13.65%**	**±2.91%**	**80.00%**	**±2.98%**

τ_m_: mean fluorescence lifetime, n: number of features, SSC: short spectral channel, LSC: long spectral channel, TP: true positive, FN: false negative, FP: false positive, TN: true negative, TPR: true positive rate, FPR: false positive rate, IR: inner ring, OR: outer ring, T1: temporal in the inner ring, S1: superior in the inner ring, S2: superior in the outer ring (ref. Figure 3A).

## Data Availability

Data underlying the results presented in this paper are not publicly available at this time but may be obtained from the authors upon reasonable request.

## Supplementary Materials

The following supporting information can be downloaded at https://www.mdpi.com/article/10.3390/diagnostics14040431/s1: Supplementary Table S1: Demographic information of all examined subjects; Supplementary Table S2: Layer-wise evaluation results on OCT-A data: non-smokers vs. all smokers; Supplementary Table S3: Layer-wise evaluation results on OCT-A data: non-smokers vs. heavy smokers (cumulative pack count ≥ 2500); Supplementary Table S4: Layer-wise evaluation results on OCT-A data: non-smokers vs. light smokers (cumulative pack count < 2500).
