# Supplementary material for: Artificial Intelligence in Fluorescence Lifetime Imaging Ophthalmoscopy (FLIO) Data Analysis—Toward Retinal Metabolic Diagnostics
Source: Diagnostics (Basel). 2024 Feb 16;14(4):431. doi: 10.3390/diagnostics14040431 (PMC10888399; doi:10.3390/diagnostics14040431)
Supplement: Supplementary file 1 [file diagnostics-14-00431-s001.zip › Supp. Table S1_corr.pdf]

ID	Smoking	Sex (0: female, 1: male)	Age	Weight (kg)	Height (cm)	Iris color	Lens	Cigarette/ day	Smoking year	Pack Year (py)	Cumulative pack number
1	non-smoker	female	28	65	174	brown	phakic	0	0	0	0
2	non-smoker	female	23	69	170	green	phakic	0	0	0	0
3	non-smoker	female	29	68	170	blue	phakic	0	0	0	0
4	non-smoker	male	26	73	177	blue	phakic	0	0	0	0
5	non-smoker	female	29	60	174	blue	phakic	0	0	0	0
6	non-smoker	female	25	60	160	green	phakic	0	0	0	0
7	non-smoker	male	24	95	191	brown, green	phakic	0	0	0	0
8	non-smoker	male	25	85	187	brown	phakic	0	0	0	0
9	non-smoker	male	21	65	176	brown	phakic	0	0	0	0
10	non-smoker	female	23	50	160	brown	phakic	0	0	0	0
11	non-smoker	male	31	95	180	green	phakic	0	0	0	0
12	non-smoker	female	24	65	173	blue	phakic	0	0	0	0
13	non-smoker	female	22	61	173	blue	phakic	0	0	0	0
14	non-smoker	male	28	89	184	brown	phakic	0	0	0	0
15	non-smoker	female	21	73	175	blue	phakic	0	0	0	0
16	non-smoker	female	30	64	165	blue	phakic	0	0	0	0
17	non-smoker	male	22	93	191	blue	phakic	0	0	0	0
18	non-smoker	male	24	73	190	brown	phakic	0	0	0	0
19	non-smoker	male	22	65	180	brown	phakic	0	0	0	0
20	non-smoker	male	33	104	196	brown	phakic	0	0	0	0
21	non-smoker	female	27	61	163	green	phakic	0	0	0	0
22	non-smoker	male	35	74	193	blue	phakic	0	0	0	0
23	non-smoker	female	27	54	160	blue	phakic	0	0	0	0
24	non-smoker	male	32	83	184	green	phakic	0	0	0	0
25	non-smoker	female	32	47	158	brown	phakic	0	0	0	0
26	non-smoker	male	31	85	194	green	phakic	0	0	0	0
27	smoker	female	25	50	155	blue	phakic	7	10	3.5	1277.5
28	smoker	female	34	105	196	brown	phakic	12.5	15	9.4	3421.9
29	somker	male	24	88	197	blue	phakic	8	5	2.0	730.0
30	smoker	female	22	63	175	blue	phakic	7	6	2.1	766.5
31	smoker	male	29	65	170	green	phakic	3	10	1.5	547.5
32	smoker	male	26	76	179	green	phakic	20.5	12	12.3	4489.5
33	somker	male	20	89	194	blue	phakic	10	4.5	2.3	821.3
34	smoker	male	30	112	200	brown	phakic	8	14.5	5.8	2117.0
35	smoker	female	25	71	163	green	phakic	15	11	8.3	3011.3
36	smoker	female	24	58	160	brown	phakic	10	9	4.5	1642.5
37	somker	male	25	75	187	green	phakic	20	9.5	9.5	3467.5
38	smoker	female	37	52	154	brown	phakic	12.5	20	12.5	4562.5
39	smoker	male	35	115	177	green	phakic	25	17	21.3	7756.3
40	smoker	male	27	193	68	brown	phakic	15	12	9.0	3285.0
41	somker	female	26	100	166	blue, green	phakic	5	5	1.3	456.3
42	smoker	male	35	74.6	179	blue	phakic	22	19	20.9	7628.5
43	smoker	female	32	95	163	brown	phakic	8	16	6.4	2336.0
44	smoker	male	30	90	182	blue	phakic	5.5	12	3.3	1204.5
45	somker	male	25	86	173	brown	phakic	6	8	2.4	876.0
46	smoker	male	24	70	173	brown	phakic	20	10	10.0	3650.0
47	smoker	female	24	90	163	blue	phakic	22.5	8	9.0	3285.0
48	smoker	female	36	66.5	168	brown	phakic	13.5	23	15.5	5666.6
49	somker	male	32	112	190	blue, green	phakic	25	17	21.3	7756.3
50	smoker	male	36	64	167	brown	phakic	12.5	20	12.5	4562.5
51	smoker	male	30	95	186	blue	phakic	9	10.5	4.7	1724.6
52	smoker	female	26	77	159	brown	phakic	5.5	9	2.5	903.4
53	somker	female	29	75	163	green	phakic	5	9	2.3	821.3
54	smoker	female	31	85	180	blue	phakic	12.5	12.5	7.8	2851.6
