# Supplementary material for: Artificial Intelligence in Fluorescence Lifetime Imaging Ophthalmoscopy (FLIO) Data Analysis—Toward Retinal Metabolic Diagnostics
Source: Diagnostics (Basel). 2024 Feb 16;14(4):431. doi: 10.3390/diagnostics14040431 (PMC10888399; doi:10.3390/diagnostics14040431)
Supplement: Supplementary file 1 [file diagnostics-14-00431-s001.zip › Supp. Table S2.pdf]

Supp. Table S2: Layer-wise evaluation results on OCT-A data: non-smokers vs. all smokers

Layer	Mean TP	Mean FN	Mean FP	Mean TN	Mean TPR	Mean FPR	Mean Accuracy
Full	29.5	24.5	32.4	19.6	54.63% ± 5.48%	62.31% ± 6.27%	46.32% ± 3.81%
Vitreoretinal Interface	39.6	14.4	31.85	20.15	73.33% ± 6.21%	61.25% ± 4.18%	56.37% ± 3.53%
Retina	28.2	25.8	26.55	25.45	52.22% ± 3.64%	51.06% ± 7.63%	50.61% ± 3.92%
SVC	21.25	32.75	27.9	24.1	39.35% ± 6.17%	53.65% ± 5.80%	42.78% ± 3.42%
NFLVP	35.7	18.3	33.6	18.4	66.11% ± 6.14%	64.62% ± 4.14%	51.04% ± 2.67%
SVP	19.45	34.55	28.25	23.75	36.02% ± 6.04%	54.33% ± 5.44%	40.75% ± 4.13%
DVC	35.65	18.35	22.65	29.35	66.02% ± 5.66%	43.56% ± 5.58%	61.32% ± 4.37%
ICP	24.85	29.15	29.25	22.75	46.02% ± 4.03%	56.25% ± 4.78%	44.91% ± 2.47%
DCP	38.1	15.9	18.1	33.9	70.56% ± 4.64%	34.81% ± 6.96%	67.92% ± 3.40%
Avascular Complex	34.95	19.05	28.3	23.7	64.72% ± 5.02%	54.42% ± 4.43%	55.33% ± 3.38%
CC	33.55	20.45	26	26	62.13% ± 3.91%	50.00% ± 2.98%	56.18% ± 2.88%
Choroid	31.95	22.05	29.65	22.35	59.17% ± 5.81%	57.02% ± 3.86%	51.23% ± 3.44%
HL	35.7	18.3	37.65	14.35	66.11% ± 7.90%	72.40% ± 5.21%	47.22% ± 4.18%
ILMtoBM	31.9	22.1	27.4	24.6	59.07% ± 3.70%	52.69% ± 4.10%	53.30% ± 2.50%
SL	31.65	22.35	34	18	58.61% ± 9.30%	65.38% ± 4.47%	46.84% ± 4.01%

Full: all layers, SVC: supeficial vasular complex, NVLVP: nerve fiber layer vascular plexus, SVP: superficial vascular plexus, DVC: deep vascular complex, ICP: intermediate capillary plexus, DCP: deep capillary plexus, avascular complex, CC: choriocapillaris, choroid, HL: Haller's layer, ILMtoBM: internal limiting membrane to Bruch membrane, SL: Sattlers's layer
