# Supplementary material for: Artificial Intelligence in Fluorescence Lifetime Imaging Ophthalmoscopy (FLIO) Data Analysis—Toward Retinal Metabolic Diagnostics
Source: Diagnostics (Basel). 2024 Feb 16;14(4):431. doi: 10.3390/diagnostics14040431 (PMC10888399; doi:10.3390/diagnostics14040431)
Supplement: Supplementary file 1 [file diagnostics-14-00431-s001.zip › Supp. Table S3.pdf]

Supp. Table S3: Layer-wise evaluation results on OCT-A data: non-smokers vs. heavy smokers (cumulative pack count ≥ 2,500)

Layer	Mean TP	Mean FN	Mean FP	Mean TN	Mean TPR	Mean FPR	Mean Accuracy
Full	11.15	16.85	22.3	29.7	39.82% ±7.51%	42.88% ±4.75%	51.06% ±4.11%
Vitreoretinal Interface	18.6	9.4	21.05	30.95	66.43% ±6.53%	40.48% ±4.57%	61.94% ±3.15%
Retina	9.15	18.85	24.35	27.65	32.68% ±6.42%	46.83% ±5.41%	46.00% ±4.14%
SVC	10.1	17.9	18.2	33.8	36.07% ±7.40%	35.00% ±4.62%	54.87% ±4.14%
NFLVP	14.25	13.75	18.55	33.45	50.89% ±6.48%	35.67% ±4.73%	59.63% ±3.01%
SVP	9.7	18.3	17.6	34.4	34.64% ±9.18%	33.85% ±4.65%	55.12% ±4.16%
DVC	11.8	16.2	18.25	33.75	42.14% ±6.74%	35.10% ±3.84%	56.94% ±3.70%
ICP	6.1	21.9	21.85	30.15	21.79% ±7.04%	42.02% ±6.03%	45.31% ±5.12%
DCP	14.25	13.75	14.05	37.95	50.89% ±7.73%	27.02% ±4.01%	65.25% ±3.48%
Avascular Complex	13.35	14.65	18.05	33.95	47.68% ±6.51%	34.71% ±3.41%	59.13% ±2.41%
CC	9.6	18.4	22.55	29.45	34.29% ±5.80%	43.37% ±3.77%	48.81% ±3.17%
Choroid	10.4	17.6	16.35	35.65	37.14% ±6.33%	31.44% ±4.05%	57.56% ±4.43%
HL	9.6	18.4	24.3	27.7	34.29% ±5.46%	46.73% ±5.44%	46.62% ±4.35%
ILMtoBM	16.5	11.5	18.65	33.35	58.93% ±4.72%	35.87% ±5.21%	62.31% ±3.86%
SL	12	16	21.95	30.05	42.86% ±6.39%	42.21% ±4.92%	52.56% ±4.32%

Full: all layers, SVC: supeficial vasular complex, NVLVP: nerve fiber layer vascular plexus, SVP: superficial vascular plexus, DVC: deep vascular complex, ICP: intermediate capillary plexus, DCP: deep capillary plexus, avascular complex, CC: choriocapillaris, choroid, HL: Haller's layer, ILMtoBM: internal limiting membrane to Bruch membrane, SL: Sattlers's layer
