# Supplementary material for: Artificial Intelligence in Fluorescence Lifetime Imaging Ophthalmoscopy (FLIO) Data Analysis—Toward Retinal Metabolic Diagnostics
Source: Diagnostics (Basel). 2024 Feb 16;14(4):431. doi: 10.3390/diagnostics14040431 (PMC10888399; doi:10.3390/diagnostics14040431)
Supplement: Supplementary file 1 [file diagnostics-14-00431-s001.zip › Supp. Table S4.pdf]

Supp. Table S4: Layer-wise evaluation results on OCT-A data : non-smokers vs. light smokers (cumulative pack count < 2,500)

Layer	Mean TP	Mean FN	Mean FP	Mean TN	Mean TPR	Mean FPR	Mean Accuracy
Full	0	26	0.4	51.6	0.00% ± 0.00%	0.77% ± 1.86%	66.15% ± 1.24%
Vitreoretinal Interface	1.25	24.75	2.45	49.55	4.81% ± 3.82%	4.71% ± 2.31%	65.13% ± 2.31%
Retina	0	26	0.7	51.3	0.00% ± 0.00%	1.35% ± 1.73%	65.77% ± 1.15%
SVC	0.65	25.35	0.85	51.15	2.50% ± 3.05%	1.63% ± 1.64%	66.41% ± 1.75%
NFLVP	0.55	25.45	2.1	49.9	2.12% ± 2.85%	4.04% ± 3.84%	64.68% ± 2.73%
SVP	0.65	25.35	1.65	50.35	2.50% ± 2.79%	3.17% ± 2.38%	65.38% ± 2.07%
DVC	0.15	25.85	3.1	48.9	0.58% ± 1.37%	5.96% ± 2.42%	62.88% ± 1.49%
ICP	0	26	1.35	50.65	0.00% ± 0.00%	2.60% ± 1.95%	64.94% ± 1.30%
DCP	0	25	3.3	48.7	3.85% ± 3.85%	6.35% ± 1.73%	63.72% ± 1.72%
Avascular Complex	1.8	24.2	2.7	49.3	6.92% ± 2.88%	5.19% ± 2.36%	65.51% ± 1.76%
CC	0.05	25.95	2.95	49.05	0.19% ± 0.84%	5.67% ± 6.10%	62.95% ± 3.99%
Choroid	0	26	1.35	50.65	0.00% ± 0.00%	2.60% ± 1.10%	64.94% ± 0.73%
HL	0	26	0.2	51.8	0.00% ± 0.00%	0.38% ± 0.77%	66.41% ± 0.51%
ILMtoBM	0	26	0.2	51.8	0.00% ± 0.00%	0.38% ± 1.30%	66.41% ± 0.87%
SL	0	26	0.3	51.7	0.00% ± 0.00%	0.58% ± 1.73%	66.28% ± 1.15%

Full: all layers, SVC: supeficial vasular complex, NVLVP: nerve fiber layer vascular plexus, SVP: superficial vascular plexus, DVC: deep vascular complex, ICP: intermediate capillary plexus, DCP: deep capillary plexus, avascular complex, CC: choriocapillaris, choroid, HL: Haller's layer, ILMtoBM: internal limiting membrane to Bruch membrane, SL: Sattlers's layer
